# Molecular Characterization of *Lactobacillus plantarum* DMDL 9010, a Strain with Efficient Nitrite Degradation Capacity

**DOI:** 10.1371/journal.pone.0113792

**Published:** 2014-11-25

**Authors:** Yong-tao Fei, Dong-mei Liu, Tong-hui Luo, Gu Chen, Hui Wu, Li Li, Yi-gang Yu

**Affiliations:** College of Light Industry and Food Science, South China University of Technology, Guangzhou, Guangdong, China; National Center for Biotechnology Information (NCBI), United States of America

## Abstract

Nitrites commonly found in food, especially in fermented vegetables, are potential carcinogens. Therefore, limiting nitrites in food is critically important for food safety. A *Lactobacillus* strain (*Lactobacillus* sp. DMDL 9010) was previously isolated from fermented vegetables by our group, and is not yet fully characterized. A number of phenotypical and genotypical approaches were employed to characterize *Lactobacillus* sp. DMDL 9010. Its nitrite degradation capacity was compared with four other *Lactobacillus* strains, including *Lactobacillus casei* subsp. *rhamnosus* 719, *Lactobacillus delbrueckii* subsp. *bulgaricu* 1.83, *Streptococcus thermophilus* 1.204, and *lactobacillus plantarum* 8140, on MRS medium. Compared to these four *Lactobacillus* strains, *Lactobacillus* sp. DMDL 9010 had a significantly higher nitrite degradation capacity (*P<0.001*). Based on 16S rDNA sequencing and sequence comparison, *Lactobacillus* sp. DMDL 9010 was identified as either *Lactobacillus plantarum* or *Lactobacillus pentosus*. To further identify this strain, the flanking regions (922 bp and 806 bp upstream and downstream, respectively) of the L-lactate dehydrogenase 1 (*L-ldh1*) gene were amplified and sequenced. *Lactobacillus* sp. DMDL 9010 had 98.92 and 76.98% sequence identity in the upstream region with *L. plantarum* WCFS1 and *L. pentosus* IG1, respectively, suggesting that *Lactobacillu* sp. DMDL 9010 is an *L. plantarum* strain. It was therefore named *L. plantarum* DMDL 9010. Our study provides a platform for genetic engineering of *L. plantarum* DMDL 9010, in order to further improve its nitrite degradation capacity.

## Introduction

Amongst all processed vegetables, fermented vegetables have the highest productivity and are important Asian cuisine. While fermentation has been widely used in food processing for over 2,000 years, vegetable fermentation has experienced low levels of industrialization. Additionally, the presence of high levels of salt and nitrites in fermented vegetables is a major health concern. Excessive intake of salt is harmful to human health and nitrites are potential carcinogens [1]–[2]. Therefore, understanding the degradation of salt and nitrites during the process of vegetable fermentation is critically important to food safety.

The Lactobacillus genus consists of over 180 bacterial species that are rod-shaped, gram-positive, and facultative anaerobic or microaerophilic bacteria [3]. Certain salt-tolerant *Lactobacillus* species are widely used in vegetable fermentation [4]. Our previous study demonstrated that addition of *Lactobacillus casei* subsp. *rhamnosus* 719 significantly reduced the concentration of nitrites [5], as well as salt [6], in fermented vegetables. We also isolated a *Lactobacillus* strain (strain DMDL 9010) from naturally fermented vegetables. Preliminary results suggested that the DMDL 9010 strain can inhibit the nitrite accumulation in vegetable fermentation and that the fermentation can be completed within 24 hours without the addition of salt. Detailed characterization of this strain is important to understand the underlying mechanism of the nitrite degradation, and may also facilitate its utilization in vegetable fermentation with genetic engineering. Thus, we aimed to characterize DMDL 9010, using a number of phenotypical and genotypical approaches.

With the rapid development of molecular biology, particularly DNA sequencing technologies, genotypical characterization of bacterial strains is widely utilized in research. For species identification and genotyping of bacteria, 16S rDNA sequencing, repetitive sequencing-based PCR (REP-PCR), PCR-restriction fragment length polymorphism (PCR-RFLP), and DNA-DNA hybridization are the most commonly used [7]–[11]. Over 97% and 99% of sequence identity of 16S rDNA is widely accepted to be the criteria for the determination of genus and species, respectively [12]. DNA sequencing of 16S rDNA is mostly common method for bacterial species identification, because universal primers are suitable for amplifying 16S rDNA from unknown bacterial strains and a large number of DNA sequences of 16S rDNA are available for almost all known bacterial species in GenBank. Search and sequence comparison of 16S rDNA with GenBank is very useful for species identification of bacteria. However, using DNA-DNA hybridization, Fox, *et al*., found that three *Bacillaceae* strains that shared 99.5% sequence identity to 16S rDNA belonged to different species, suggesting that 16S rDNA sequence identity may not be sufficient to guarantee species identity [13]. PCR-RFLP is usually used in genus identification of bacteria [7] and is not distinguishable when the sequence identity of 16S rDNA is higher than 96% [14]. REP-PCR has the advantages of being easy to conduct and producing a high resolution that is useful for discriminating strain sharing of over 99.5% sequence identity of 16S rDNA; however, REP-PCR has low reproducibility and is easily contaminated [15]. DNA-DNA hybridization is considered to be the golden standard for species identification of bacteria, and a 70% cut-off is most often used to place organisms into different species [16]. However, the DNA-DNA hybridization protocol is time-consuming and a large amount of genomic DNA is required, which limits its application in fastidious bacteria [17]. DNA sequences with high sequence variations, such as non-coding intergenic regions, have recently been used in bacterial strain typing and molecular identification. In this study, we used both highly conserved 16S rDNA sequences and two non-coding fragments, in order to characterize DMDL 9010.

## Materials and Methods

### Bacterial strains

*Lactobacillus* sp. DMDL9010 was isolated by the Food Safety Laboratory, College of Light Industry and Food, South China University of Technology, from fermented vegetables in 2011 [18]. It was first identified as *Lactobacillus pentosus* DMDL 9010 and stored in China General Microbiological Culture Collection (CGMCC) (storage #:5172). *Lactobacillus casei* subsp. *rhamnosus* 719 was stored in the Food Safety Laboratory, College of Light Industry and Food, South China University of Technology. *Lactobacillus delbrueckii* subsp. *bulgaricus* 1.83 and *Streptococcus thermophiles* 1.204 were purchased from Guangdong Institute of Microbial Culture Collection (GIMCC) and *Lactobacillus plantarum* 8140 was purchased from CGMCC.

### Measurement of nitrites

According to the GB/T5009.33-2010 reference on "measurement of food nitrite and nitrate", a naphthyl ethylenediamine hydrochloride assay was used to measure nitrites, albeit with slight modifications. Briefly, without the protein precipitation step, 2 ml sulfanilic acid was added into the solution, mixed, and left to stand 3∼5 min. Then, 1 ml naphthyl ethylenediamine solution (2 g/L) was added to reach to designated volume, mixed, and left to stand for 15 min. The absorbance was then measured, in order to determine the concentration of nitrites.

### Degradation of nitrites by *Lactobacillus* sp. DMDL 9010

10 ml sterilized MRS medium (Guangdong Haikou Microbiology Biotech Inc., Haikou, China), containing 10.00 mg/L NaNO_2_ was added into 15 ml sterilized test tube. Next, 5% (v/v) *Lactobacillus* sp. DMDL 9010 starter was added to the test tube and sealed. The solution was cultured at 37°C for 24 h. 1 ml fermented sample was collected, sterilized, and tested, as mentioned above. Each fermentation sample was measured for nitrites three times. The concentration of nitrites is presented as mean value ± standard deviation.

### Molecular identification and characterization of *Lactobacillus* sp. DMDL9010

*Lactobacillus* sp. DMDL 9010 was first identified by sequencing 16S rDNA. Overnight cultured bacterial strains (0.1–1.5 ml) were centrifuged at 12000 (r/min) at room temperature for 1 min to precipitate cell pellets. Next, 0.6 ml bacterial lysozyme was added, mixed, and kept at 37°C for 40 min to break down bacterial cells. Genomic DNA was isolated using the DNA isolation kit (Takara, Dalian, China), according to the manufacturer's protocol. Isolated genomic DNA was used as a template in PCR amplification of 16S rDNA. Primers used for amplification of 16S rDNA are F8 (forward): 5′-AGA GTT TGA TCC TGG CTC AG-3′ and R1492 (reverse): 5′-TAC GGT TAC CTT GTT ACG ACT-3′. PCRs were carried out in a PTC-200 automated thermal cycler (Bio-Rad CO., LTD). One nanomolar concentration of each DNA preparation was amplified in a 25-µl reaction mixture containing 50 pM of each primer, 200 µM (each) dATP, dCTP, dGTP, and dTTP (Takara, Dalian, China), as well as 0.125 µl *Taq* polymerase (Takara, Dalian, China) and an appropriate volume of distilled water. PCR conditions consisted of an initial denaturation at 95°C for 5 min and 30 cycles of 30 s at 94°C, 30 s at 56°C, and 2 min at 72°C, with a final extension at 72°C for 7 min. PCR products were analyzed by electrophoresis in a 1.5% agarose gel and purified using a purification DNA fragment kit Ver 2.0 (Takara, Dalian, China). The PCR products were then sent to Takara Biotechnology (Takara, Dalian, China) for DNA sequencing. The determined DNA sequences of 16S rDNA were searched in the GenBank database using the BLAST program. Top hits with known representative species were selected and sequences were downloaded and aligned using the Clustal W program (www.ebi.ac.uk/Tools/msa/clustalw2). The alignment was trimmed and imported into the Mega 5.1 program to construct the phylogenetic organization using the Neighbor-Joining method.

Since the 16S rDNA is highly conserved between closely related species, we used another marker of high sequence diversity to further characterize *Lactobacillus* sp. DMDL9010. The gene encoding L-lactate dehydrogenase 1 (*L-ldh1*) is a housekeeping gene that is highly conserved in bacteria. We chose two fragments flanking *L-ldh1* as molecular markers to identify this strain. Primers used to amplify these two markers were F1 (forward): 5′-TATCCGTACTGTGTTTCCTC-3′, R1 (reverse): 5′-ACTAGAACCAACAGCGCCGT-3′, F2 (forward): 5′-TAGGTGGCCTTTTCGGTAGC-3′, and R2 (reverse): 5′-CTCGTCTATAGCAGACGGGC-3′. The PCR reaction system was the same for amplifying 16S rDNA. To amplify the up-stream fragment using F1 and R1 primers, PCR conditions consisted of an initial denaturation at 95°C for 5 min and 30 cycles of 30 s at 94°C, 30 s at 58°C, and 1.5 min at 72°C, with a final extension of 72°C for 7 min. In order to amplify the down-stream fragment using the F1 and R1 primers, PCR conditions consisted of an initial denaturation at 95°C for 5 min and 30 cycles of 30 s at 94°C, 30 s at 59°C, and 1.5 min at 72°C, with a final extension of 72°C for 7 min. Electrophoresis of PCR products, as well as their DNA sequencing, were conducted in the same way as for the 16S rDNA mentioned above. The determined DNA sequences of these two fragments were searched in the GenBank database using the BLAST program, to identity the most similar sequences and species. The results are combined with phenotypical results and 16S rDNA sequence similarity to determine the species of *Lactobacillus* sp. DMDL 9010.

## Results and Discussion

### *Lactobacillus* sp. DMDL9010 has the highest capacity of nitrite degradation

**Figure 1:**
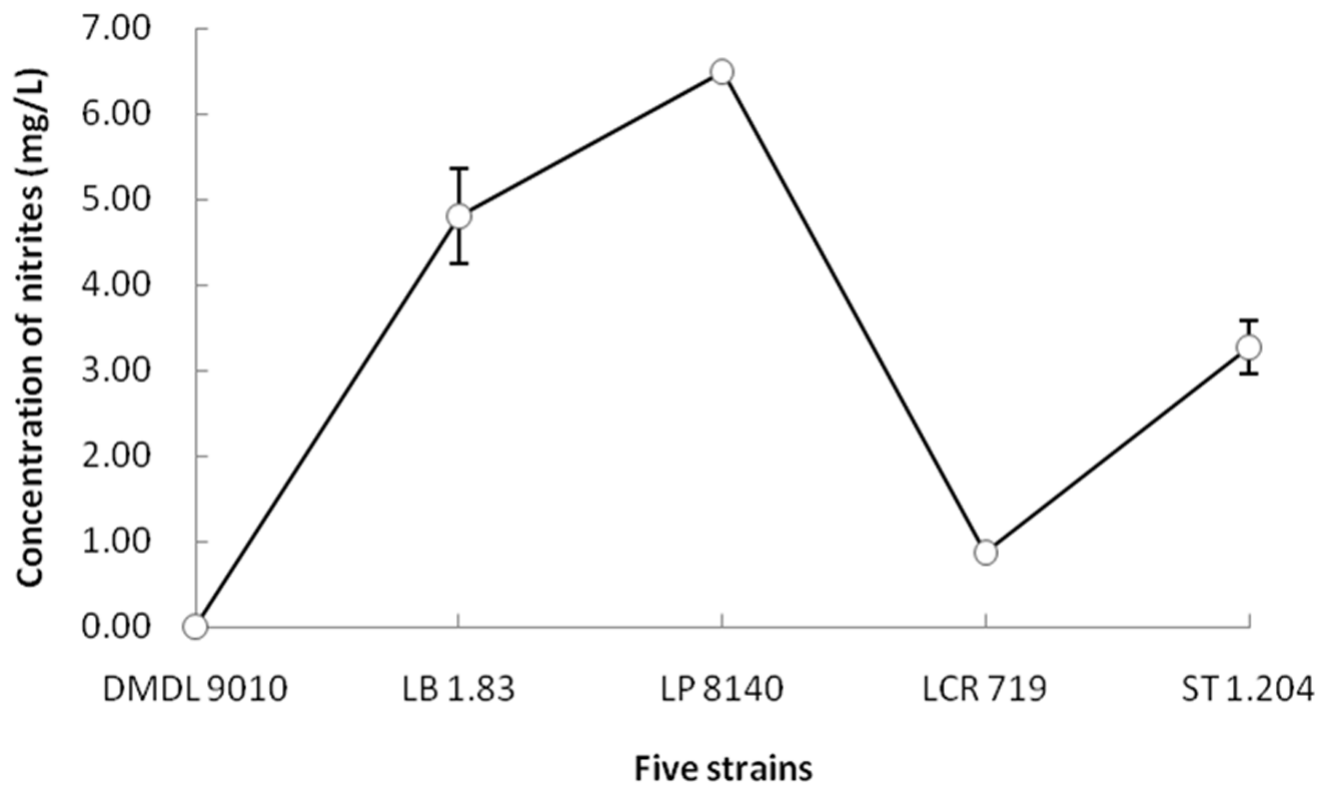
Comparison of the effects of nitrite degradation among five different *Lactobacillus* strains in the MRS medium. After fermentation for 24 h at 37°C, the nitrite concentrations in the MRS medium were 0.00 mg/L, 4.80±0.56 mg/L, 6.49±0.05 mg/L, 0.87±0.05 mg/L, 3.27±0.31 mg/L, and for strains DMDL 9010, LB 1.83, LP 8140, LCR 719 and ST 1.204, respectively. Compared to the other four strains, *Lactobacillus* sp. DMDL 9010 showed the highest capacity of nitrite degradation (*p<0.001*).

As shown in Figure 1, after 24 hours of fermentation, the nitrite concentrations in the MRS medium were 0.00±0.00 mg/L, 4.80±0.56 mg/L, 6.49±0.05 mg/L, 0.87±0.05 mg/L, and 3.27±0.31 mg/L for strains DMDL 9010, LB 1.83, LP 8140, LCR 719and ST 1.204, respectively. *Lactobacillus* sp. DMDL 9010 degraded nitrites (10 mg/L) in the MRS medium to a level not detectable after 24 hours of fermentation (Figure 1). Among the other four strains, LP 8140 exhibited the lowest capacity of nitrite degradation. Compared to the other four strains, *Lactobacillus* sp. DMDL 9010 showed the highest capacity of nitrite degradation (*p<0.001*).

### Lactobacillus sp. DMDL9010 is *Lactobacillus plantarum* or *Lactobacillus pentosus*, based on the 16S rDNA sequence

**Figure 2:**
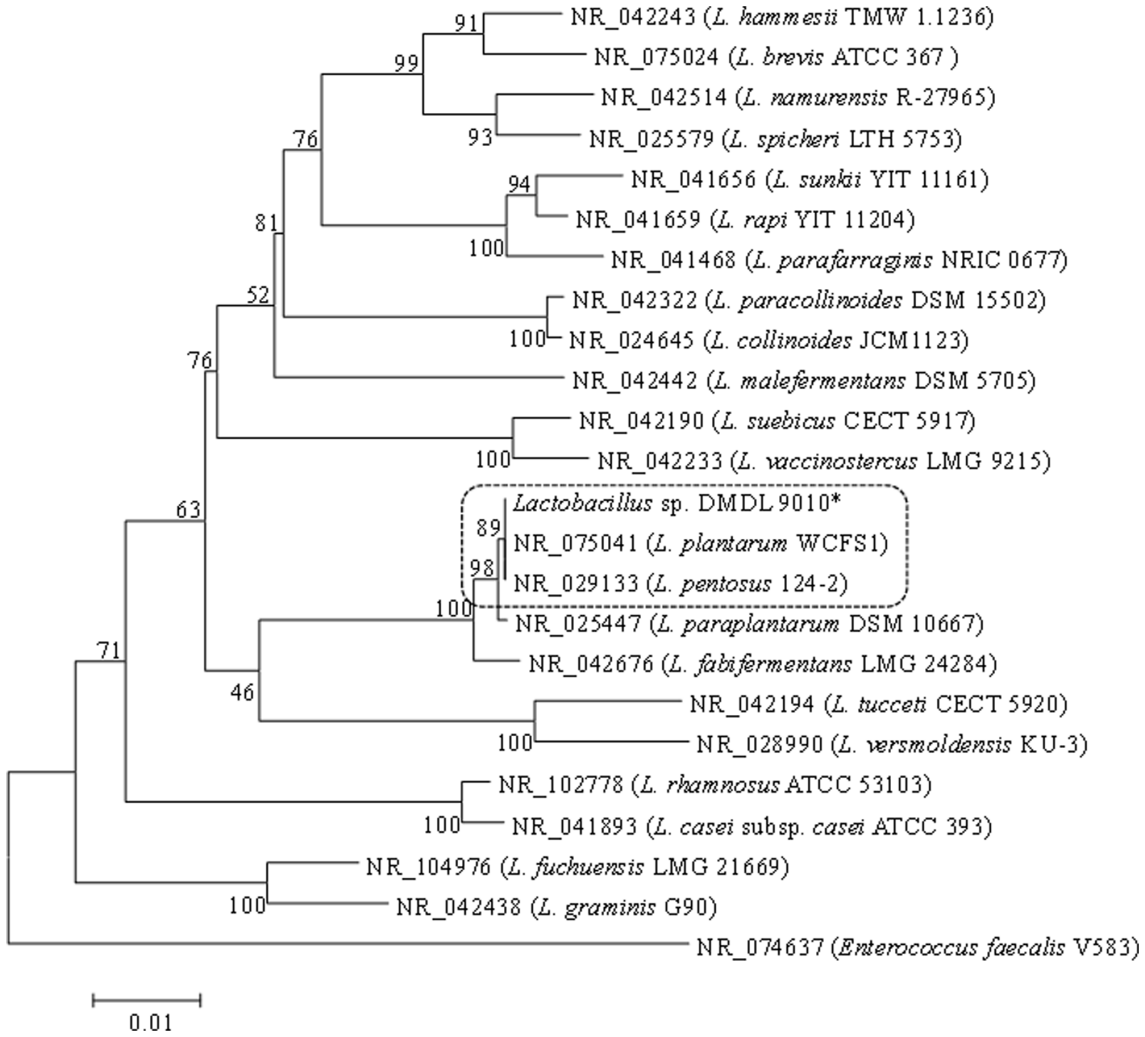
Phylogenetic organization of *Lactobacillus* species and *Lactobacillus* sp. DMDL 9010, based on 16S rDNA sequences. The DNA sequences of different *Lactobacillus* species were retrieved from GenBank and aligned with the 16S rDNA sequence of *Lactobacillus* sp. DMDL 9010 using Clustal W. The “NR” numbers are GenBank accession numbers of *Lactobacillus* species. Phylogenetic organization was obtained using the neighbor-joining and maximum parsimony methods within the MEGA 5 software. Bootstrap values based on 500 replicates were shown at the nodes of the phylogenetic tree. The bar represents 0.01 nucleotide changes.

**Figure 3:**
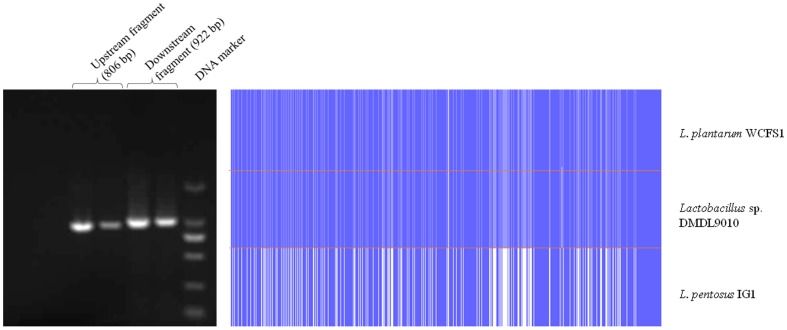
PCR amplification of the upstream and downstream regions of the *L-ldh1* gene from *Lactobacillus* sp. DMDL 9010. A 922 bp section upstream from the *L-ldh1* gene and an 806 bp fragment downstream were amplified from *Lactobacillus* sp. DMDL 9010. Sequence alignment of the upstream region of the *L-ldh1* gene among *Lactobacillus* sp. DMDL 9010, *L. plantarum* WCFS1, and *L. pentosus* IG1. Blue and blank bands represent regions of high and low sequence identity, respectively. The sequence alignment shows that *Lactobacillus* sp. DMDL 9010 is more closely related to *L. plantarum* than *L. pentosus*, since *Lactobacillus* sp. DMDL 9010 and *L. plantarum* share 98.92% sequence identity, which is much higher than the 76.98% sequence identity between *Lactobacillus* sp. DMDL 9010 and *L. plantarum* WCFS1.

A PCR amplicon of 1500 bp was successfully amplified from *Lactobacillus* sp. DMDL 9010 (Figure 2). Finally, a 1441 bp fragment was obtained from DNA sequencing and was deposited in the GenBank database (accession number: KJ 917253). Based BLASTn search in GenBank using this 1441 bp fragment as the query, we found that the 16S rDNA of *Lactobacillus* sp. DMDL 9010 share 99% sequence identity with *Lactobacillus plantarum* and *Lactobacillus pentosus*. We then retrieved 22 DNA sequences of 16S rDNA from different *Lactobacillus* species from GenBank for phylogenetic analysis. The phylogenetic analysis based on these 16S rDNA suggested that *Lactobacillus* sp. DMDL9010 is closely related to *Lactobacillus plantarum* and *Lactobacillus pentosus* (Figure 3). Therefore, 16S rDNA sequences are not suitable for discriminating *L. pentosus* and *L. plantarum* species, because of high sequence identity. A number of genes and non-coding sequences have been used for species identification of bacteria. For example, *rpoB* sequences have been shown to be powerful for characterization of *Corynebacterium* at the species level (PMID: 15364970). Non-coding sequences are typically more variable than housekeeping genes and can be used for discrimination of closely related strains and species. Therefore, in order to further classify *Lactobacillus* sp. DMDL 9010, we sequenced the flanking regions of the *L-ldh1*gene.

### Carbohydrate fermentation of *Lactobacillus* sp. DMDL 9010

Based on the physiological and biochemical characteristics [18]–[19] and the sequence comparison of the 16S rRNA gene, *Lactobacillus* sp. DMDL 9010 has been determined to be of the *Lactobacillaceae* phylum, *Lactobacillales* class, *Bacilli* order, and *Firmicutes* family, according to the “Berger Bacterial Identification Handbook" and "Bergey's Manual of Systematic Bacteriology [20].” To further phenotypically characterize this strain, a carbohydrate fermentation of *Lactobacillus* sp. DMDL9010 was performed. Carbohydrate fermentation was conducted using 17 types of carbohydrates, including arabinose, cellobiose, fructose, galactose, glucose, maltose, mannose, rhamnose, ribose, sorbose, sucrose, trehalose, xylose, raffinose, and esculin (Table 1). The carbohydrate fermentation results showed that *Lactobacillus* sp. DMDL 9010 is capable of utilizing galactose, mannose, glucose, maltose, sucrose, trehalose, xylose, raffinose, and esculin (Table 1). However, the application to other carbohydrates was insufficient to further discriminate *L. pentosus* and *L. plantarum*, and we therefore decided to sequence more polymorphic DNA, in order to discriminate the two.

**Table 1:** Carbohydrate fermentation of strain DMDL 9010.

Carbohydrate	arabinose	cellobiose	fructose	galactose	glucose	maltose	maltose mannose	rhamnose	ribose	sorbose	sucrose	trehalose	xylose	raffinose	esculin	
Fermentation result																

(“+”represent positive, “-” represent negative)

### The sequence flanking the *L-ldh1* gene suggests that *Lactobacillus* sp. DMDL 9010 is *Lactobacillus plantarum*

Two fragments consisting of 922 and 806 bp were amplified from *Lactobacillus* sp. DMDL 9010, using *L-ldh1* upstream primers (5′-TATCCGTACTGTGTTTCCTC-3′ and 5′-ACTAGAACCAACAGCGCCGT-3′) and downstream primers (5′-TAGGTGGCCTTTTCGGTAGC-3′ and 5′-CTCGTCTATAGCAGACGGGC-3′) (Figure 3). These two fragments were sequenced and deposited in the GenBank database (accession numbers: KM 017408 and KM 017409, respectively). The top hits of the fragments were from *L. plantarum*, suggesting that *Lactobacillus* sp. DMDL 9010 is closely related to *L. plantarum*. Based on sequence alignment using Clustal W, identity of the upstream sequence was 98.92% between *L. plantarum* WCFS1 and *Lactobacillus* sp. DMDL 9010, but was 76.98% between *L. pentosus* IG1 and *Lactobacillus* sp. DMDL 9010. The significantly higher sequence identity of upstream of *L-ldh1* between *Lactobacillus* sp. DMDL 9010 and *L. plantarum* than *L. pentosus* suggests that the upstream region of *L-ldh1* can be used for discrimination between *L. plantarum* and *L. pentosus* when 16S rDNA fails to identify between the two species.

No DNA sequences downstream of *L. pentosus L-ldh1* are available in GenBank. Genomic sequences of *L. pentosus* strains in GenBank are incomplete and gaps were found around in the downstream of *L-ldh1*. Therefore, sequence comparison of the downstream region of *L-ldh1* was not conducted between *L. pentosus* and our strain of *Lactobacillus* sp. DMDL 9010. The sequence identity downstream from *L-ldh1* was 99.54% between *L. plantarum* WCFS1 and *Lactobacillus* sp. DMDL 9010. This dramatically high sequence identity of the downstream region from *L-ldh1* suggests that *Lactobacillus* sp. DMDL 9010 is closely related to *L. plantarum* WCFS1. Taken together, the sequence similarity of the upstream region of *L-ldh1* between *Lactobacillus* sp. DMDL 9010 and *L. plantarum* was much high than that between *Lactobacillus* sp. DMDL 9010 and *L. pentosus*, suggesting *Lactobacillus* sp. DMDL9010 is more closely related to *L. plantarum*. Combined with the results of 16S rDNA sequencing and phenotypical characterization, *Lactobacillus* sp. DMDL 9010 is considered to be an *L. plantarum* strain and was named *L. plantarum* DMDL 9010.

## Conclusion

In this study, we characterized a *Lactobacillus* strain that was previously isolated from fermented vegetables and exhibited significant nitrite degradation capability, using a number of phenotypical and genotypical approaches, including physiological and biochemical characterization, 16S rDNA sequencing, and DNA sequencing of flanking regions of the *L-ldh1* gene. Our results demonstrated that *Lactobacillus* sp. DMDL 9010 has the higher nitrite degradation capability than other four *Lactobacillus* strains we examined, by degrading nitrites in the MRS fermentation medium to an undetectable level. Based on sequence analysis of 16S rDNA and the flanking regions of the *L-ldh1* gene, this strain was determined to be *L. plantarum* DMDL 9010. We plan to sequence the entire genome of *L. plantarum* DMDL 9010, which will certainly improve our understanding of its evolution and metabolic pathways, including nitrite degradation. The genomic sequence may also facilitate genetic engineering of this strain to further utilize its role in vegetable fermentation.
